# To Mars and beyond. Space exploration and space medicine in *Leukemia*

**DOI:** 10.1038/s41375-024-02286-8

**Published:** 2024-05-22

**Authors:** R. P. Gale, Andreas Hochhaus

**Affiliations:** 1 Centre for Haematology, Department of Immunology and Inflammation, Imperial College of Science, Technology and Medicine, SW7 2AS London, UK; 2 Klinik für Innere Medizin II, Universitätsklinikum Jena, Jena, Germany

**Keywords:** Cancer, Health care

Humans are curious. We have explored every aspect of Earth from the top of Everest (8849 m) to the bottom of the Challenger Deep (11035 m), from the North to the South Poles and everywhere in between. Explorers risked their lives trying to reach these destinations, many died en route. On Magellan’s global circumnavigation only 35 of 243 sailors returned. Vasco de Gama on his trip from Portugal to India did a bit better but still only 55 of 170 sailors made it to the end. No one survived Scott’s South Pole expedition; Shackleton’s expedition was luckier.

The next frontier is Space. Men have landed on the Moon, lived in the International Space Station (ISS) and are likely to set out for Mars within this decade. One commonly overlooked aspect of Space exploration is the potential effect of astronauts’ exposure to ionizing radiations. For example, a round-trip journey to Mars is estimated to last about 5 years. Space explorers will have to pass through the Van Allen radiation belts encircling Earth and will then be exposed to ionizing radiations from diverse sources within our Solar System and from the Cosmos including charged particles and ultraviolet rays from the Sun, galactic cosmic rays (GCRs), charged particles originating outside our solar system and trapped by planetary magnetic fields. These exposures will increase as we attempt to inhabit other planets. A solar coronal mass ejection (CME) would expose people living on the Moon to about 1 Sv of radiation, enough to cause acute radiation syndrome.  One just happened  revealing the aurora borealis to most of the Northern Hemisphere.

Most haematologists are aware of the increased cancer risk associated with exposure to ionizing radiation the most serious of whiche is excess leukaemias, lymphomas and plasma cell myeloma. Risks of solid cancers ares also increased. However, it is unlikely haematologists ey are thinking about this issue in the context of Space exploration.

We think now is the time to raise awareness of the potential haematological effects of Space exploration which are not limited to radiation but also effects of micro-gravity on bone marrow function. As such we present readers with 2 Perspectives on Space medicine which we hope these will raise awareness, peak interest and, perhaps most importantly given the long timeline of Space exploration, encourage young haematologists to consider a career in Space medicine [1, 2].

Robert Peter Gale and Andreas Hochhaus, Editors-in-Chief.
