# Correction: To Mars and beyond. Space exploration and space medicine in *Leukemia*

**DOI:** 10.1038/s41375-024-02324-5

**Published:** 2024-06-25

**Authors:** Robert Peter Gale, Andreas Hochhaus

**Affiliations:** 1 Centre for Haematology, Department of Immunology and Inflammation, Imperial College of Science, Technology and Medicine, SW7 2AS London, UK; 2 Klinik für Innere Medizin II, Universitätsklinikum Jena, Jena, Germany

**Keywords:** Cancer, Health care

Correction to: *Leukemia* 10.1038/s41375-024-02286-8, published online 22 May 2024

In this article refs. [1, 2] were incorrect and should have been:
